# A commentary on the updated research priorities in ophthalmology: implications and future directions

**DOI:** 10.1038/s41433-026-04263-0

**Published:** 2026-03-05

**Authors:** Rupert R. A. Bourne, Malik Moledina, Augusto Azuara-Blanco, George M. Saleh, James Self, Sobha Sivaprasad, Srilakshmi M. Sharma, Andrew Ross, Rose M. Gilbert, Maram E. A. Abdalla Elsayed, Won Young Moon, Manjo Doug, Pádraig J. Mulholland, Alex C. Day, Vito Romano, Geraldine V. Hoad, Madina Kara, Martin Cordiner, Louise Gow, Faruque Ghanchi, Praveen J. Patel, Richard Gale, Christiana Dinah, Keith Valentine, Cathy Yelf, Vanessa Poustie, NIHR Ophthalmology Specialty Group, Ejaz Ansari, Jane Ashworth, Madeline Bayne, Nick AV Beare, Rupert RA Bourne, Philip Buckhurst, Hilary Campbell, Emma Chambers, Fransesco Cordeiro, Joanne Creighton, Christiana Dinah, Manjo Doug, Susan Downes, Declan Flanagan, Richard Gale, Faruque Ganchi, Irene Gottlob, Louise Gow, Chris Hammond, Geraldine V. Hoad, Jonathan Jackson, Sarah Kennedy, Anthony King, Andrew J. Lotery, Geeta Menon, Sarah Moll, Padraig J Mulholland, Ian Nickson, Praveen Patel, Vanessa Poustie, Fiona J. Rowe, George Saleh, Peter Scanlon, Brinda Shah, Julie Silvestri, Pelota Sung, Mihaela Sutu, Andrew Tatham, El Kashab Tarek, Marta Urgarte, Keith Valentine, Deepali Varma, Marcela Vortruba, Saila Waseem, Executive Group of UK Clinical Eye Research Strategy, Rupert RA Bourne, Manjo Doug, Richard Gale, Faruque Ganchi, Louise Gow, Geraldine V. Hoad, Praveen Patel, Vanessa Poustie, George Saleh, Deepali Varma, Augusto Azuara-Blanco, Michael Bowen, Catey Bunce, Nick Caplin, Roxanne Crosby, Ali Ghareeb, Renata Gomes, Kerry Hanna, Tina Houlihan, Madina Kara, Liying Low, Ailish Murray, James Self, Srilakshmi Sharma, Sobha Sivaprasad

**Affiliations:** 1 Vision and Eye Research Institute, School of Medicine, Anglia Ruskin University, Cambridge, UK; 2 Department of Ophthalmology, Cambridge University Hospitals, Cambridge, UK; 3 Imperial College NHS Foundation Trust, London, UK; 4 Centre for Public Health, Queen’s University Belfast, Belfast, UK; 5 Research, Cataract and Adnexal Department, Moorfields Eye Hospital, London, UK; 6 University of Southampton, Southampton, UK; 7 NIHR Moorfields Clinical Research Facility, Moorfields Eye Hospital, London, UK; 8 Dept of Ophthalmology, Oxford Eye Hospital, Oxford University Hospitals NHS Trust, Oxford, UK; 9 Aswan University Hospital, Aswan, Egypt; 10 NIHR Biomedical Research Centre at Moorfields Eye Hospital NHS Foundation Trust and UCL Institute of Ophthalmology, London, UK; 11 Centre for Optometry & Vision Sciences, School of Biomedical Sciences, Ulster University, Coleraine, Northern Ireland UK; 12 Department of Medical and Surgical Specialties, Radiological Sciences, and Public Health, University of Brescia, Brescia, Italy; 13 Macular Society, Andover, UK; 14 Fight for Sight, London, UK; 15 Moorfields Eye Charity, Moorfields Eye Hospital, London, UK; 16 Eye care and living well services, Royal National Institute of Blind people, London, UK; 17 Bradford Teaching Hospitals, Royal Infirmary, Bradford, West Yorkshire UK; 18 York Hospital, University of York, York, UK; 19 Department of Ophthalmology, London North West University Healthcare NHS Trust, London, UK; 20 Brain Sciences, Imperial College London, London, UK; 21 NIHR Clinical Research Network, University of Liverpool, Liverpool, UK; 22 Maidstone and Tunbridge Wells NHS Trust, Maidstone, UK; 23 Manchester University NHS Foundation Trust, Manchester, UK; 24 NHS Research Scotland, Edinburgh, UK; 25 Eye and Vision Science, University of Liverpool, Liverpool, UK; 26 University of Plymouth, Plymouth, UK; 27 York and Scarborough Teaching Hospitals NHS Foundation Trust, York, UK; 28 CRN National Coordinating Centre, Leeds, UK; 29 Imperial College Healthcare NHS Trust, London, UK; 30 Glaucoma UK, Ashford, UK; 31 Oxford Eye Hospital, Oxford, UK; 32 Moorfields Eye Hospital, London, UK; 33 Bradford Teaching Hospitals NHS Foundation Trust, Bradford Royal Infirmary, Bradford, UK; 34 University of Leicester, Leicester, UK; 35 Guy’s and St Thomas’ NHS Foundation Trust, London, UK; 36 Belfast Health and Social Care Trust, Belfast, UK; 37 The University of Nottingham, Nottingham, UK; 38 Faculty of Medicine, University of Southampton, Southampton, UK; 39 Frimley Health NHS Foundation Trust, Surrey, UK; 40 NIHR Clinical Research Network Yorkshire and Humber, Leeds, UK; 41 NIHR Clinical Research Network Coordinating Centre, University of Liverpool, Liverpool, UK; 42 Institute of Population Health, University of Liverpool, Liverpool, UK; 43 Gloucestershire Hospitals NHS Foundation Trust, Gloucester, UK; 44 Somerset NHS Foundation Trust, Taunton, UK; 45 Birmingham and Midland Eye Centre, Birmingham, UK; 46 University College London, London, UK; 47 Princess Alexandra Eye Pavilion and University of Edinburgh, Edinburgh, UK; 48 Mid Cheshire Hospitals NHS Foundation Trust, Crewe, UK; 49 Manchester University NHS Foundation Trust and University of Manchester, Manchester, UK; 50 Sunderland Eye Infirmary, South Tyneside and Sunderland NHS Foundation Trust, Sunderland, UK; 51 University Hospital Wales and Cardiff University, Cardiff, UK; 52 The College of Optometrists, London, UK; 53 Royal Marsden NHS Foundation Trust, London, UK; 54 Blind Veterans UK, London, UK; 55 NIHR Trainee Research Network Representative (Northern region), London, UK; 56 BIOS Research, University of Reading, Reading, UK; 57 Retina UK, London, UK; 58 Department of Ophthalmology, Oxford Eye Hospital, Oxford University Hospitals NHS Trust, Oxford, UK

**Keywords:** Outcomes research, Education

The recently published study that refreshed the James Lind Alliance Sight Loss and Vision Priority Setting Partnership research priorities [1] represents a significant effort in shaping the future of ophthalmic research by the UK Clinical Eye Research Strategy (UKCERS) [2]. While the study effectively re-evaluated the most pressing research needs in ophthalmology, this commentary highlights additional considerations and implications of these findings, particularly in light of emerging research and advancements in the field.

The study’s methodology ensured diverse stakeholder engagement, providing valuable insights into the evolving landscape of ophthalmic research. The emphasis on patient and professional involvement enhances the credibility of the prioritisation process. However, there remain key areas that warrant further attention.

The study does not explicitly address the role of artificial intelligence (AI) and precision medicine in ophthalmology. Recent advances in AI-driven diagnostics, machine learning models for disease progression prediction and personalised treatment approaches could significantly impact areas such as early detection of visual disorders and improved treatments for glaucoma and age-related macular degeneration. Integrating these aspects into future research priorities is crucial for optimising patient outcomes [3].

The survey results highlight the importance of integrating ophthalmic primary and secondary care via community optometric pathways. However, disparities in access to ophthalmic care persist, particularly among underserved populations. Future research should focus on evaluating barriers to care, the impact of telemedicine in ophthalmology and the development of cost-effective screening and treatment programs for at-risk groups.

While the study effectively identifies current priorities, there is a need for robust longitudinal studies that track the long-term effectiveness of interventions. Additionally, harnessing real-world data through national registries and electronic health records could provide valuable insights into disease progression and treatment efficacy across diverse populations.

Following the publication of the updated research priorities, the UKCERS team has developed Population, Intervention, Comparison and Outcome (PICO) frameworks to further refine and operationalise these priorities. The PICO model provides a structured approach to formulating precise and actionable research questions that align with the identified needs in ophthalmology.

By applying the PICO framework, we can:

- Clearly define the patient populations most affected by specific conditions, ensuring targeted research.
- Specify key interventions and compare them to existing or alternative treatment modalities.
- Establish measurable outcomes that can guide the development of clinical trials and observational studies.

*Example 1: Addressing the priority area of research into the treatment of age-related macular degeneration (AMD). A PICO-based approach might frame a research question as follows:*

- Population: Patients with intermediate AMD.
- Intervention: The Valeda Light Delivery System (photobiomodulation therapy) or oral medications (e.g. metformin, AREDS multivitamin formulation, sirolimus, L-dopa, tonabersat, SGLT2 inhibitors, fenofibrate, statins).
- Comparison: Standard care with no intervention or placebo.
- Outcome: Slowed progression to late-stage AMD (neovascular or geographic atrophy) and preservation of visual function.

*Example 2: Addressing the priority area of research into Ophthalmic biomarkers of early Alzheimer’s disease. A PICO-based approach might frame a research question as follows:*

- Population: Adults under assessment for *Alzheimer’s disease*
- Intervention: A comprehensive visual assessment which might include; posterior segment OCT imaging, fundus photography, eye movement assessment +/- visual processing or other techniques combined into one visual assessment tool.
- Comparison: NHS standard Alzheimer’s diagnostic criteria (‘Revised Criteria for Diagnosis and Staging of Alzheimer’s Disease’ was published June 27, 2024 in Alzheimer’s & Dementia)
- Outcome: Diagnostic accuracy. Sensitivity and specificity of the visual assessments to identify people with Alzheimer’s disease.

By incorporating these and other specific PICOs into future research initiatives, we can ensure that research priorities are systematically developed into focused and actionable studies that can drive meaningful advances in eyecare. This is of particular importance given that eye research remains underfunded when compared to its societal impact.

Vision loss is estimated to affect two million people in the UK, costing the economy £25 billion annually [https://www.google.com/url?q=https://www.sightresearchuk.org/eye-research/our-research/&source=gmail-imap&ust=1732711886000000&usg=AOvVaw1dSnCzMIYV6VLum5IJZR9m]. Despite this, UK funding for eye research represents only 1.5% of medical research spending, a fraction of what is allocated to areas such as cancer or cardiovascular research [https://www.openaccessgovernment.org/article/sight-loss-research-equitable-future/163563/]. This disparity places the UK at risk of falling behind nations like the USA, where the National Eye Institute invests over $800 million annually in vision research. In order to redress this imbalance, the Clinical Study Groups of UKCERS have prepared several PICOs across different subspecialty areas of Ophthalmology for submission to UK’s National Institute for Health Research (NIHR). The intention is to lead to commissioned calls for research into these specific areas among the NIHR’s nine research programmes that fund multi-disciplinary health and social care research in areas that include clinical evaluation and translation, health services and organisation, technology development, public health and social care.

The updated research priorities offer a valuable framework for advancing ophthalmology research. Integration of emerging technologies, equitable access to care and long-term data-driven approaches need also to be emphasised in future iterations of priority setting. Additionally, applying the PICO framework ensures that research priorities are systematically developed into focused and actionable studies. By addressing these additional considerations, ophthalmology research can be further aligned with evolving clinical needs and technological advancements, ultimately improving patient care and outcomes.
